# Mutational and immunogenetic landscape of HCV‐associated B‐cell lymphoproliferative disorders

**DOI:** 10.1002/ajh.26167

**Published:** 2021-04-09

**Authors:** Irene Defrancesco, Marcella Visentini, Silvia Zibellini, Ylenia Aura Minafò, Sara Rattotti, Virginia Valeria Ferretti, Ettore Rizzo, Marzia Varettoni, Marco Frigeni, Alessandro Pulsoni, Milvia Casato, Stefania Colantuono, Marianna Rossi, Chiara Candido, Caterina Zerbi, Fabio Bergamini, Caterina Cristinelli, Nicole Fabbri, Michele Merli, Valentina Zuccaro, Raffaele Bruno, Marco Paulli, Luca Arcaini

**Affiliations:** ^1^ Department of Medical, Surgical, Diagnostic and Pediatric Sciences University of Pavia Pavia Italy; ^2^ Division of Hematology Fondazione IRCCS Policlinico San Matteo Pavia Italy; ^3^ Department of Translational and Precision Medicine Sapienza University of Rome Rome Italy; ^4^ Laboratory affiliated to Istituto Pasteur Italia ‐ Fondazione Cenci Bolognetti Sapienza University of Rome Rome Italy; ^5^ Department of Molecular Medicine Sapienza University of Rome Rome Italy; ^6^ Clinical Epidemiology and Biometrics Unit Fondazione IRCCS Policlinico San Matteo Pavia Italy; ^7^ enGenome srl Pavia Italy; ^8^ Department of Molecular Medicine University of Pavia Pavia Italy; ^9^ Division of Hematology, University Hospital "Ospedale di Circolo e Fondazione Macchi"‐ASST Sette Laghi University of Insubria Varese Italy; ^10^ Division of Infectious Diseases Fondazione IRCCS Policlinico San Matteo Pavia Italy; ^11^ Division of Anatomic Pathology Fondazione IRCCS Policlinico San Matteo Pavia Italy; ^12^ Now at Istituto Dermopatico dell'Immacolata, IDI‐IRCCS Rome Italy; ^13^ Now at Division of Hematology, Azienda Socio‐Sanitaria Territoriale Papa Giovanni XXIII Bergamo Italy

To The Editor:

Besides robust epidemiological evidences, the direct link between HCV and B‐cell lymphoproliferative disorders (LPDs) has been sustained by clinical studies that showed lymphoma regression after HCV eradication.^1^, ^2^ However, data regarding molecular characteristics of HCV‐associated LPDs are still limited so far. The main purpose of our study was to explore the mutational profile of 27 patients with previously untreated HCV‐associated low‐grade LPDs by means of an extensive NGS genes panel.

Seven and twenty patients were diagnosed and managed at the Division of Hematology, Fondazione IRCCS Policlinico San Matteo, Pavia, Italy and at the Reference Center for Mixed Cryoglobulinemia, University “La Sapienza”, Rome, Italy, respectively. For all patients, either peripheral blood (PB) (n = 19) or bone marrow (BM) (n = 6) samples or formalin‐fixed paraffin‐embedded (FFPE) tissue (n = 2) obtained at the time of LPD diagnosis were available (online supplemental methods). Clinical and virological data were retrospectively collected. The study was approved by the Ethics Committees of the Fondazione IRCCS Policlinico San Matteo, Pavia, Italy and of Sapienza University, Rome, Italy.

Immunoglobulin heavy variable (IGHV) and light variable chain (IGLV) genes rearrangements were assessed using the IGH Somatic Hypermutation Assay v2.0 kit (Invivoscribe, San Diego, California) or according to the BIOMED‐2 guidelines. All IGH, IGK (κ light chain) and IGL (λ light chain) rearrangements were analyzed using the IMGT databases and the IMGT/V‐QUEST tool to identify CDR3 AA sequences. Heavy chain CDR3 (HCDR3) and light chain CDR3 (LCDR3) stereotypy and homology to anti‐HCV E2 antibodies and rheumatoid factors (RF) were searched, as previously described in 13 patients.^3^Targeted NGS analysis (144 genes panel) was performed by a probe‐capture based strategy, as we previously described^4^ (Table S1). The average depth of coverage was 1700x. Bioinformatics analysis was performed by an ad hoc pipeline, as already reported.^5^

Quantitative variables were summarized as median and range. Qualitative variables were described as counts and relative frequencies of each category. Association between two categorical variables was evaluated by Fisher's exact test. Mann–Whitney test was used to compare a quantitative variable among two independent groups of patients. Overall Survival (OS) was calculated as the time between diagnosis and death for any cause or last follow‐up. Progression‐Free Survival (PFS) was defined as the time between diagnosis and the date of progression, or death or last follow‐up. OS and PFS were estimated by Kaplan–Meier product‐limit method. Note, *p* values < .05 were considered significant. Statistical analyses were performed by Stata 16 (StataCorp. 2019. Stata Statistical Software: Release 16, College Station, TX: StataCorp LLC).

Clinical and virological characteristics of patients with indolent B‐cell non‐Hodgkin's lymphomas (B‐NHLs) (n = 22) and type II mixed cryoglobulinemia (MC) (n = 5) are listed in Table S2. After a median follow‐up of 4.3 years (range 0.3–25.2), the 5‐year OS was 71% (95% CI 41.6%–87.5%) and 5‐year PFS was 66.9% (95% CI 40%‐83.8%).

Data on productive IGHV rearrangements were available for 24 patients (Figure S1 and Table S3). So, IGHV1‐69 was the most used rearrangement (6, 25%), especially in type II MC (4/5, 80%). IGLV rearrangement was studied in 15 cases, revealing strong gene usage bias, with *Vk3D‐20* as the most frequent (6/15, 40%) and preferential pairing with IGHV1‐69 in all cases but one. The KCDR3 sequences were stereotyped in 10 out of 14 cases, all treated with direct‐acting antiviral agents (DAA) (Table S3). Patients with stereotyped KCDR3s showed a higher number of hematological responses to AT compared with non‐stereotyped KCDR3 group (60% vs 0%, respectively; difference between risks 60%, 95% CI: 30%–90%; *p* = .044).

The NGS analysis revealed 85 somatic mutations in 42 genes with at least one mutation (median three, range 1–8) in 25 cases (Figure 1, Panel A). Two patients with type II MC did not show mutations. Overall, the most frequently mutated genes were *TNFAIP3* (7, 28%), *KMT2C* (5, 20%), *FAT4* (5, 20%), *TBL1XR1* (5, 20%), *FAT1* (4, 16%), *CARD11* (3, 12%), *KLF2* (3, 12%), *KLHL6* (3, 12%), *PTPRD* (3, 12%). Of note, patients with type II MC did not harbor recurrent mutations of *KMT2C*, *FAT4*, *TBL1XR1*, *CARD11* genes. Full annotation and variant allele frequencies for each mutation are reported in Table S4.

**FIGURE 1:**
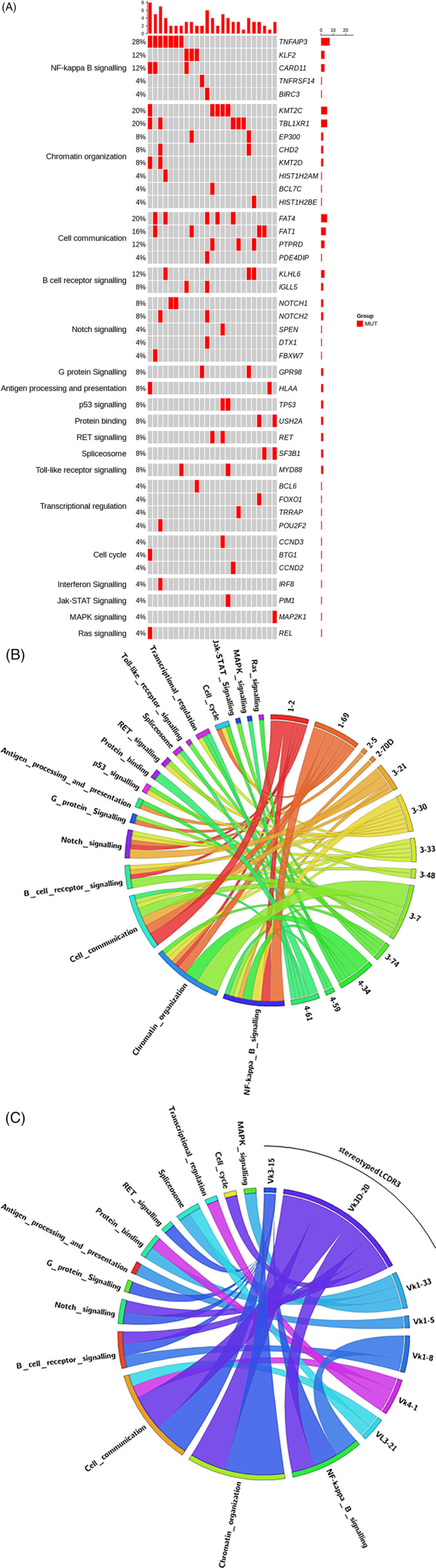
Pattern of somatic mutations in 27 patients with B cell lymphoproliferative disorders associated to hepatitis C virus. (A), Prevalence of driver genetic lesions according to the involved pathway; (B), Circos plots representing the relative frequencies and pairwise co‐occurrence of the involved pathways according to IGHV usage; (C), Circos plots representing the relative frequencies and pairwise co‐occurrence of the involved pathways and IGLV rearrangements according to light chains CDR3 sequences

Overall, genes regulating chromatin organization were the most mutated (48.2%), followed by genes belonging to NF‐kB signaling (44.4%), cell communication (40.7%), NOTCH (22.2%) and BCR (18.5%) signaling pathways (Figure S2).

Remarkably, while IGHV status did not significantly differ according to involved pathway (Figure 1, Panel B), the Vκ3D‐20 and Vκ3‐15 subsets of stereotyped KCDR3 were enriched in mutations involving chromatin organization, NF‐kB, BCR and NOTCH signaling pathways compared to the patients with non‐stereotyped KCDR3s (*p* = .002). Conversely, the latter group was highly enriched (*p* < .001) in sporadically mutated genes involving transcriptional regulation, MAPK, and protein‐binding signaling pathways (Table S5). The relative frequencies of pathways and IGLV rearrangements according to LCDR3s are illustrated in Figure 1 (Panel C).

In this study we performed an extended NGS analysis to characterize 27 HCV‐positive LPDs.

Interestingly, we found that pathways regulating epigenetics were the most involved, in line with the fact that HCV might modulate epigenetics in hepatocellular carcinoma (HCC) tumorigenesis.^6^ It is also noteworthy that the aforementioned pathways, together with NF‐kB, BCR and NOTCH signaling pathways, were more frequently mutated in patients harboring stereotyped LCDR3s, who also showed a higher number of hematological responses to AT. These findings suggest that a subset of HCV‐related LPDs may harbor distinct molecular features as well as specific immunogenetic signature, as recently reported,^3^ further supporting the concept that specific stereotyped B‐cell receptors (BCRs) may promote or select oncogenic mutations in LPDs, as already proposed in chronic lymphocytic leukemia.

We recognized that our data suffer from some limitations: we could not confirm the somatic origin of mutations because of the lack of control samples; moreover, the retrospective nature of the study, together with the relatively small size of the series, the heterogeneity of LPDs and the diverse follow‐up, limited us to provide significant associations between clinical and molecular features.

In summary, to the best of our knowledge, this was the first study that explored by means of an extensive targeted NGS panel the mutational profile of HCV‐associated B‐cell LPDs, identifying recurrently mutated genes and pathways. Our results demonstrate also that a subset of HCV‐related LPDs may harbor distinct molecular and immunogenetic features and bring out the potential correlation between specific BCR configurations, genetic lesions and regression of the lymphoproliferation after HCV eradication. A clinical trial with DAAs in indolent HCV‐positive lymphomas is ongoing with ancillary biological studies and will provide further data in this setting (NCT02836925).

## CONFLICT OF INTEREST

None of the authors has relevant conflicts of interest related to the content of this work.

L.A. received advisory honoraria from Roche, Celgene, Janssen‐Cilag, Verastem, Eusa Pharma, and Incyte, research support from Gilead, and travel expenses from Roche, Celgene, Janssen‐Cilag, and Eusa Pharma. E.R. has shares of enGenome srl, an Italian bioinformatics company. A.P. was an advisory board member for Roche, Merk, Pfizer, Sandoz, and Takeda and a speaker for Roche, Gilead, and Bristol Myers Squibb. M.V. received advisory honoraria from Janssen, Roche, Astra Zeneca and travel expenses from AbbVie. R.B. was an advisory board member and a speaker for AbbVie, Gilead Sciences and Merck Sharp & Dohme.

## AUTHOR CONTRIBUTIONS

L.A. conceived, designed, and supervised the study; S.Z., C.C., M.F., E.R., performed IGHV and NGS analyses in Pavia; M.Vi. and Y.A.M. performed IGLV and IGHV analyses in Rome; M.Vi., A.P., M.C., S.C., I.D., S.R., M.R., C.Z., F.B., C.C., N.F. M.V., M.M., V.Z., R.B., M.P. acquired clinical data; E.R. performed bioinformatics analysis; L.A., I.D., V.V.F., S.Z, and M.Vi. analyzed and interpreted data and wrote the manuscript; and all authors revised and approved the final version of the manuscript.

## Supporting information


**Appendix S1** Supporting information

## Data Availability

The data that support the findings of this study are available from the corresponding author upon reasonable request.
