# Supplementary material for: Mutational and immunogenetic landscape of HCV‐associated B‐cell lymphoproliferative disorders
Source: Am J Hematol. 2021 Apr 9;96(6):E210–4. doi: 10.1002/ajh.26167 (PMC8252412; doi:10.1002/ajh.26167)
Supplement: Supplementary file 1 — Appendix S1 Supporting information [file AJH-96-E210-s001.docx]

# Supplemental data

**Supplemental Methods**

## Samples collection

Mononuclear cells (MNCs) were separated from BM in 6 cases and PB in 19 cases by standard density gradient centrifugation (Lympholyte-H; CEDARLANE Laboratories Ltd). Genomic DNA and RNA were extracted using standard protocols for human tissue according to the manufacturer's recommendations. RNA was extracted from cells lysed with TRIzol® Reagent and reverse transcribed. Quality and quantity of DNA and RNA were tested through NanoDrop ND-1000 Spectrophotometer or Qubit 3.0 Fluorometer (Life Technologies, Carlsbad, California, USA). In two patients genomic DNA was extracted from FFPE tissue according to standard methods.

# Supplemental Figures Legends

***Supplemental Figure 1 – Immunoglobulin heavy chain variable region gene (IGHV) rearrangements in 27 patients with hepatitis C virus-associated lymphoproliferative disorders*** HCV-associated low grade B-NHLs showed IGHV3-7 rearrangement in 3 cases out of 18 (17%), and IGHV1-69, IGHV3-30, IGHV3-33 and IGHV4-61 rearrangements each in 2 cases (11%). IGHV1-69 was the most used rearrangement among type II MC cases (80%).

***Supplemental Figure 2 –Pathways involved in 27 patients with hepatitis C virus-associated lymphoproliferative disorders***

**Supplemental Tables**

## Supplemental Table 1 - List of genes and assigned pathways included in the 144-NGS genes panel.

In two cases, NGS analysis was ran on genomic DNA extracted from FFPE tissue and these cases were sequenced with a larger panel comprising 6 additional genes highlighted in bold in the genes’ list.

## Supplemental Table 2- Clinical characteristics of 27 patients with HCV-associated B-cell lymphoproliferative disorders

*MZL: 2 splenic MZL, 1 subcutaneous extranodal MZL

^#^type II MC: 1b in 3 cases, 2a/2c in two cases; low grade NHL: 1b in 11 cases, 1a in one case, 2a/2c in 3 cases, 3a in one case

^§^HCV-RNA was positive in all patients, HCV-RNA quantitative not available in 3 patients

^Data were available for 17 patients with lymphoma, 16 received DAA-based antiviral therapy (1 CR with IFN)

°this patient was not subsequently treated and died of lung carcinoma

*Abbreviations:* type II MC=type II mixed cryoglobulinemia; B-NHL= B cell non-Hodgkin lymphoma; NOS=not otherwise specified; CLL/SLL=chronic lymphocytic leukemia/small lymphocytic lymphoma; FL=follicular lymphoma; MZL=marginal zone lymphoma; MoC=monoclonal component; IFN=interferon; DAA= direct acting antivirals; CR=complete response; PR=partial response; SD=stable disease; PD=progressive disease

## Supplemental Table 3- IGHV and IGLV rearrangements in 27 patients with HCV-associated lymphoproliferative disorders.

IGHV sequences with a percentage of identity to germline genes >98% were considered unmutated. All but 4 sequences carried somatic hypermutations (SHM) with a median identity to germline of 97.57% (range: 90.28-99.47%). Stereotyped HCDR3 motifs were identified in 4 patients (2 of them even sharing an identical IGHV1-2/D7-27/J4 sequence). IGLV rearrangements were studied in 15 cases; the overall median SHM load was 98% (range 88.35-99.33%), and 14/15 (93%) cases expressed kappa CDR3s (KCDR3). At variance with the scarce stereotypy observed among HCDR3s, similar to a previous study^1^, we identified two KCDR3 stereotyped sequences accounting overall for 66% (10/15) of light chain CDR3, a finding that might suggest a predominant role for light chains in shaping B-cell receptor specificity in HCV-associated lymphoproliferative disorders.

*unproductive

*Abbreviations:* B-NHL NOS=B cell non-Hodgkin lymphoma not otherwise specified; CLL/SLL=chronic lymphocytic leukemia/small lymphocytic lymphoma; FL= follicular lymphoma; MZL=marginal zone lymphoma; SMZL=splenic marginal zone lymphoma; type II MC= type II mixed cryoglobulinemia; E2 hom=E2 homology; RF hom=RF homology; NA=not available; DAA=direct-acting antivirals; IFN=interferon.

## Supplemental Table 4- Full annotation and variant allele frequencies for each mutation in 27 patients with hepatitis C virus-associated lymphoproliferative disorders

^*^CD19+ selection was not performed from PB or BM samples and VAFs were adjusted for the proportion of clonal CD19+ B-cells in each sample to account for tumor representation.

^°^Based on the information retrieved from the KEGG and REACTOME databases, each gene was assigned to a specific pathway.

*Abbreviations:* B-NHL NOS=B cell non-Hodgkin lymphoma not otherwise specified; CLL/SLL=chronic lymphocytic leukemia/small lymphocytic lymphoma; FL= follicular lymphoma; MZL=marginal zone lymphoma; SMZL=splenic marginal zone lymphoma; type II MC= type II mixed cryoglobulinemia.

## Supplemental Table 5- Correlation of LCDR3 stereotypes with mutational status.

Groups include patients with characterized IGHV and IGLV rearrangementsand with at least one mutated gene among 144 investigated (n=13). Mutated genes are evidenced in blue.Statistical analysis by Fisher’s exact test. We identified 3 subsets based on LCDR3 stereotypy and on homologies with anti-HCV E2 antibodies and/or rheumatoid factors (RF). Subset A (n=4) expressed a Vκ3D-20 stereotyped LCDR3 of 9 AA (paired with IGHV1-69 in 3 cases) homologous both to anti-E2 and to RF, which shared a consensus sequence with KCDR3s described in stereotypy subsets of CLL^2^; a fifth patient (EF09) with a 9 AA LCDR3 harboring the dual RF/anti-E2 homology characteristic of this subset was excluded from statistical analysis because the KCDR3 did not fulfil criteria for stereotypy. Subset B (n=4) expressed a Vκ3-15 stereotyped LCDR3 of 10 AA homologous to RF, but not to anti-HCV E2 antibodies, quasi-identical to KCDR3 sequences found in HCV-associated LPDs^3^. Subset C (n=4) expressed non-stereotyped LCDR3s lacking anti- HCV E2 antibodies (4/4) and RF (3/4) homologies, possibly representing cases of NHL casually associated with rather than dependent on HCV infection.

*Abbreviations:* Pt.=patient; NHL NOS=B-cell non-Hodgkin lymphoma not otherwise specified; CLL/SLL=chronic lymphocytic leukemia/small lymphocytic lymphoma; FL=follicular lymphoma;

MZL=marginal zone lymphoma; SMZL=splenic marginal zone lymphoma; type II MC=type II mixed cryoglobulinemia; RF hom=RF homology; E2 hom=E2 homology.

# Supplemental References

1. Minafò YA, Del Padre M, Cristofoletti C, et al. A stereotyped light chain may shape virus- specific B-cell receptors in HCV-dependent lymphoproliferative disorders. *Genes Immun*. 2020;21(2):131-135. doi:10.1038/s41435-020-0093-9
2. Hadzidimitriou A, Darzentas N, Murray F, et al. Evidence for the significant role of immunoglobulin light chains in antigen recognition and selection in chronic lymphocytic leukemia. *Blood*. 2009;113(2):403-411. doi:10.1182/blood-2008-07-166868
3. Hoogeboom R, van Kesse KPM, Hochstenbach F, et al. A mutated B cell chronic lymphocytic leukemia subset that recognizes and responds to fungi. *J Exp Med*. 2013;210(1):59-70. doi:10.1084/jem.20121801

## Supplemental Figure 1 – Immunoglobulin heavy chain variable region gene (IGHV) rearrangements in 27 patients with hepatitis C virus-associated lymphoproliferative disorders


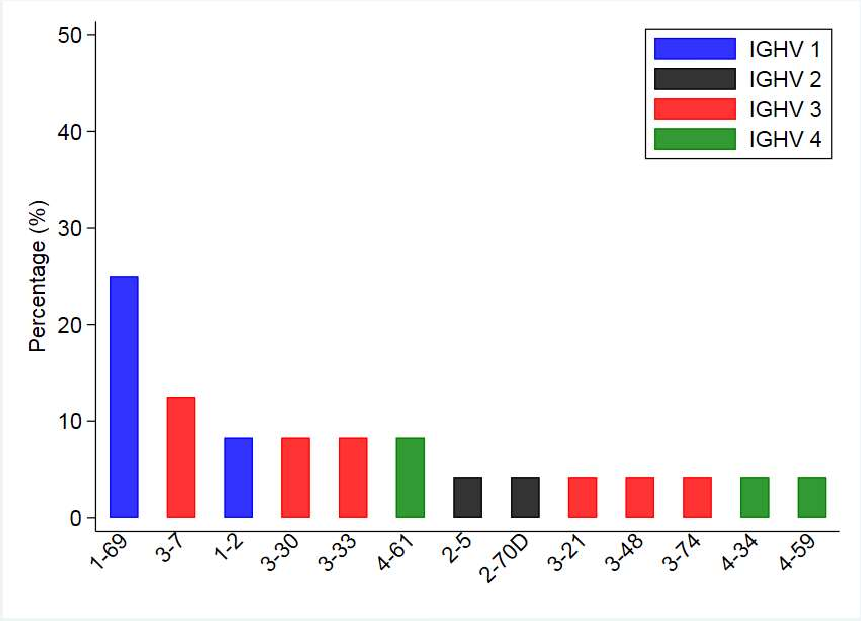


***Supplemental Figure 2 –Pathways involved in 27 patients with hepatitis C virus-associated lymphoproliferative disorders***


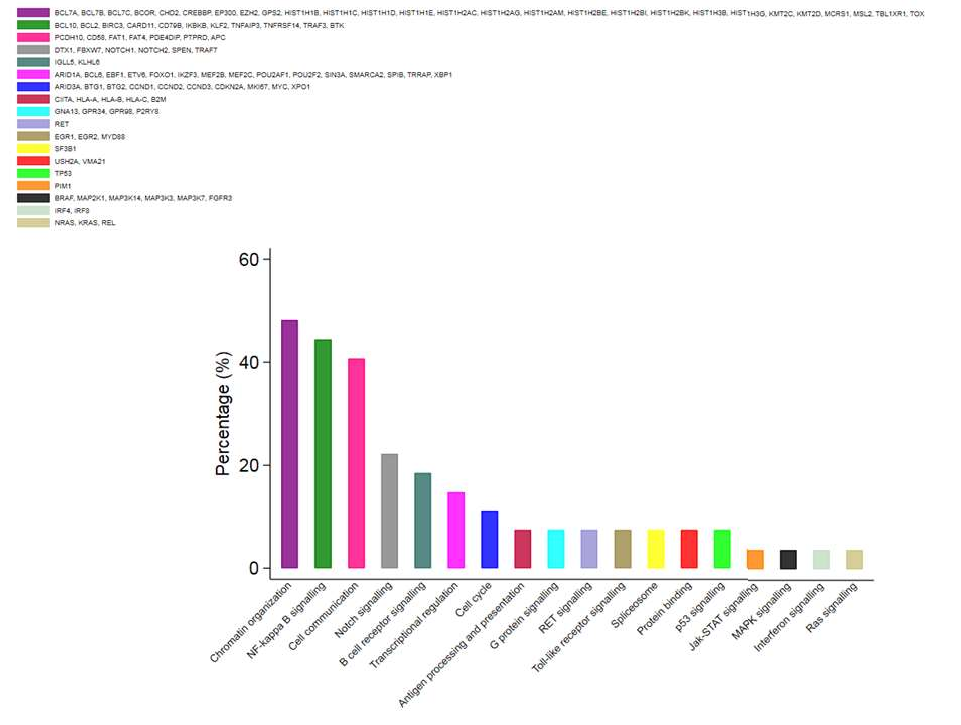


# Supplemental Table 1: List of genes and related pathways included in the NGS panel

| **GENES** | **PATHWAY** | **NCBI ID** | **CHROMOSOME LOCATION** |
| --- | --- | --- | --- |
| IGLL5 | B cell receptor signalling | 100423062 | 22q11.22 |
| KLHL6 | B cell receptor signalling | 89857 | 3q27.1 |
| CIITA | Antigen processing and  Presentation | 4261 | 16p13.13 |
| HLA-A | Antigen processing and  Presentation | 3105 | 6p22.1 |
| HLA-B | Antigen processing and  Presentation | 3106 | 6p21.33 |
| HLA-C | Antigen processing and  Presentation | 3107 | 6p21.33 |
| B2M | Antigen processing and  Presentation | 567 | 15q21.1 |
| APAF1 | Apoptosis | 317 | 12q23.1 |
| ATM | Apoptosis | 472 | 11q22.3 |
| **FAIM** | **Apoptosis** | **55179** | **3q22.3** |
| FAS | Apoptosis | 355 | 10q23.31 |
| SEMA3A | Axon guidance / Developmental  Biology | 10371 | 7q21.11 |
| PLA2G4D | Calcium ion binding | 283748 | 15q15.1 |
| PCDH10 | Cell communication | 57575 | 4q28.3 |
| CD58 | Cell communication | 965 | 1p13.1 |
| FAT1 | Cell communication | 2195 | 4q35.2 |
| FAT4 | Cell communication | 79633 | 4q28.1 |
| PDE4DIP | Cell communication | 9659 | 1q21.2 |
| PTPRD | Cell communication | 5789 | 9p24.1-p23 |
| APC | Cell communication | 324 | 5q22.2 |
| ARID3A | Cell cycle | 1820 | 19p13.3 |
| BTG1 | Cell cycle | 694 | 12q21.33 |
| BTG2 | Cell cycle | 7832 | 1q32.1 |
| CCND1 | Cell cycle | 595 | 11q13.3 |
| CCND2 | Cell cycle | 894 | 12p13.32 |
| CCND3 | Cell cycle | 896 | 6p21.1 |
| **CCNG2** | **Cell cycle** | **901** | **4q21.1** |
| CDKN2A | Cell cycle | 1029 | 9p21.3 |
| MKI67 | Cell cycle | 4288 | 10q26.2 |
| MYC | Cell cycle | 4609 | 8q24.21 |
| XPO1 | Cell cycle | 7514 | 2p15 |
| ITPKB | Cell signalling | 3707 | 1q42.12 |
| SWAP70 | Cell signalling | 23075 | 11p15.4 |
| CCR6 | Chemokine signalling | 1235 | 6q27 |
| BCL7A | Chromatin organization | 605 | 12q24.31 |
| BCL7B | Chromatin organization | 9275 | 7q11.23 |
| BCL7C | Chromatin organization | 9274 | 16p11.2 |
| BCOR | Chromatin organization | 54880 | Xp11.4 |
| CHD2 | Chromatin organization | 1106 | 15q26.1 |
| CREBBP | Chromatin organization | 1387 | 16p13.3 |

| EP300 | Chromatin organization | 2033 | 22q13.2 |
| --- | --- | --- | --- |
| EZH2 | Chromatin organization | 2146 | 7q36.1 |
| GPS2 | Chromatin organization | 2874 | 17p13.1 |
| HIST1H1B | Chromatin organization | 3009 | 6p22.1 |
| HIST1H1C | Chromatin organization | 3006 | 6p22.2 |
| HIST1H1D | Chromatin organization | 3007 | 6p22.2 |
| HIST1H1E | Chromatin organization | 3008 | 6p22.2 |
| HIST1H2AC | Chromatin organization | 8334 | 6p22.2 |
| HIST1H2AG | Chromatin organization | 8969 | 6p22.1 |
| HIST1H2AM | Chromatin organization | 8336 | 6p22.1 |
| HIST1H2BE | Chromatin organization | 8344 | 6p22.2 |
| HIST1H2BI | Chromatin organization | 8346 | 6p22.2 |
| HIST1H2BK | Chromatin organization | 85236 | 6p22.1 |
| HIST1H3B | Chromatin organization | 8358 | 6p22.2 |
| HIST1H3G | Chromatin organization | 8355 | 6p22.2 |
| KMT2C | Chromatin organization | 58508 | 7q36.1 |
| KMT2D | Chromatin organization | 8085 | 12q13.12 |
| MCRS1 | Chromatin organization | 10445 | 12q13.12 |
| MSL2 | Chromatin organization | 55167 | 3q22.3 |
| TBL1XR1 | Chromatin organization | 79718 | 3q26.32 |
| TOX | Chromatin organization | 9760 | 8q12.1 |
| CD70 | Cytokine Signalling | 970 | 19p13.3 |
| CXCR4 | Cytokine Signalling | 7852 | 2q22.1 |
| **CXCR5** | **Cytokine Signalling** | **643** | **11q23.3** |
| PIK3CD | Cytokine Signalling | 5293 | 1p36.22 |
| SOCS1 | Cytokine Signalling | 8651 | 16p13.13 |
| STAT3 | Cytokine Signalling | 6774 | 17q21.2 |
| STAT6 | Cytokine Signalling | 6778 | 12q13.3 |
| TET2 | DNA methylation | 54790 | 4q24 |
| GNA13 | G protein Signalling | 10672 | 17q24.1 |
| GPR34 | G protein Signalling | 2857 | Xp11.4 |
| GPR98 | G protein Signalling | 84059 | 5q14.3 |
| P2RY8 | G protein Signalling | 286530 | Xp22.33 and Yp11.2 |
| IRF4 | Interferon Signalling | 3662 | 6p25.3 |
| IRF8 | Interferon Signalling | 3394 | 16q24.1 |
| PIM1 | Jak-STAT Signalling | 5292 | 6p21.2 |
| BRAF | MAPK signalling | 673 | 7q34 |
| MAP2K1 | MAPK signalling | 5604 | 15q22.31 |
| MAP3K14 | MAPK signalling | 9020 | 17q21.31 |
| MAP3K3 | MAPK signalling | 4215 | 17q23.3 |
| MAP3K7 | MAPK signalling | 6885 | 6q15 |
| FGFR3 | MAPK signalling | 2261 | 4p16.3 |
| ATP6AP1 | Metabolic pathways | 537 | Xq28 |
| ATP6V1B2 | Metabolic pathways | 526 | 8p21.3 |
| RRAGC | mTOR signalling | 64121 | 1p34.3 |
| BCL10 | NF-kappa B signalling | 8915 | 1p22.3 |
| BCL2 | NF-kappa B signalling | 596 | 18q21.33 |
| BIRC3 | NF-kappa B signalling | 330 | 11q22.2 |
| CARD11 | NF-kappa B signalling | 84433 | 7p22.2 |

| CD79B | NF-kappa B signalling | 974 | 17q23.3 |
| --- | --- | --- | --- |
| IKBKB | NF-kappa B signalling | 3551 | 8p11.21 |
| **IKBKE** | **NF-kappa B signalling** | **9641** | **1q32.1** |
| KLF2 | NF-kappa B signalling | 10365 | 19p13.11 |
| TNFAIP3 | NF-kappa B signalling | 7128 | 6q23.3 |
| TNFRSF14 | NF-kappa B signalling | 8764 | 1p36.32 |
| TRAF3 | NF-kappa B signalling | 7187 | 14q32.32 |
| BTK | NF-kappa B signalling | 695 | Xq22.1 |
| **RELA** | **NF-kappa B signalling** | **5970** | **11q13.1** |
| DTX1 | Notch signalling | 1840 | 12q24.13 |
| FBXW7 | Notch signalling | 55294 | 4q31.3 |
| NOTCH1 | Notch signalling | 4851 | 9q34.3 |
| NOTCH2 | Notch signalling | 4853 | 1p12 |
| SPEN | Notch signalling | 23013 | 1p36.21-p36.13 |
| TRAF7 | Notch signalling | 84231 | 16p13.3 |
| TP53 | p53 signalling | 7157 | 17p13.1 |
| PTEN | PI3K-Akt signalling | 5728 | 10q23.31 |
| SGK1 | PI3K-Akt signalling | 6446 | 6q23.2 |
| USH2A | Protein binding | 7399 | 1q41 |
| VMA21 | Protein binding | 203547 | Xq28 |
| LRP1B | Protein transport | 53353 | 2q22.1-q22.2 |
| NRAS | Ras signalling | 4893 | 1p13.2 |
| KRAS | Ras signalling | 3845 | 12p12.1 |
| REL | Ras signalling | 5966 | 2p16.1 |
| RET | RET signalling | 5979 | 10q11.21 |
| HVCN1 | Signalling and cellular processes | 84329 | 12q24.11 |
| AMOTL1 | Signalling by GPCR | 154810 | 11q21 |
| SF3B1 | Spliceosome | 23451 | 2q33.1 |
| **CMIP** | **T cell signaling** | **80790** | **16q23** |
| ID3 | TGF-beta signalling | 3399 | 1p36.12 |
| EGR1 | Toll-like receptor signalling | 1958 | 5q31.2 |
| EGR2 | Toll-like receptor signalling | 1959 | 10q21.3 |
| MYD88 | Toll-like receptor signalling | 4615 | 3p22.2 |
| ARID1A | Transcriptional regulation | 8289 | 1p36.11 |
| BCL6 | Transcriptional regulation | 604 | 3q27.3 |
| EBF1 | Transcriptional regulation | 1879 | 5q33.3 |
| ETV6 | Transcriptional regulation | 2120 | 12p13.2 |
| FOXO1 | Transcriptional regulation | 2308 | 13q14.11 |
| IKZF3 | Transcriptional regulation | 22806 | 17q12-q21.1 |
| MEF2B | Transcriptional regulation | 100271849 | 19p13.11 |
| MEF2C | Transcriptional regulation | 4208 | 5q14.3 |
| POU2AF1 | Transcriptional regulation | 5450 | 11q23.1 |
| POU2F2 | Transcriptional regulation | 5452 | 19q13.2 |
| SIN3A | Transcriptional regulation | 25942 | 15q24.2 |
| SMARCA2 | Transcriptional regulation | 6595 | 9p24.3 |
| SPIB | Transcriptional regulation | 6689 | 19q13.33 |
| TRRAP | Transcriptional regulation | 8295 | 7q22.1 |
| XBP1 | Transcriptional regulation | 7494 | 22q12.1; 22q12 |
| FBXO11 | Ubiquitination | 80204 | 2p16.3 |

| IRF2BP2 | Ubiquitination | 359948 | 1q42.3 |
| --- | --- | --- | --- |
| MYCBP2 | Ubiquitination | 23077 | 13q22.3 |
| WAC | Ubiquitination | 51322 | 10p12.1; 10p12.1-p11.2 |
| CTNNB1 | Wnt signalling pathway | 1499 | 3p22.1 |
| DDX3X | Wnt signalling pathway | 1654 | Xp11.4 |
| LEF1 | Wnt signalling pathway | 51176 | 4q25 |

**Supplemental Table 2 - Clinical characteristics of 27 patients with HCV-associated B-cell lymphoproliferative disorders**

| **Features** | **Type II MC (n=5)** | **Low grade B-NHL (n=22)** |
| --- | --- | --- |
| Age, median (range) | 65.5 (48-78) | 65 (45-79) |
| Gender M/F | 0/5 | 10/12 |
| Diagnosis, n (%) | - | Low grade B-NHL NOS: 10 (45)  MZL*: 7 (32)  CLL/SLL: 4 (18)  FL: 1 (5) |
| Marrow involvement | - | 22 (100) |
| PB involvement, n (%) | 5 (100) | 22 (100) |
| Clonal B cells (x10^9^/L), median (range) | 0.34 (0.02-0.49) | 2.37 (0.07-74.73) |
| Cryoglobulins, n (%) | 5 (100) | 16/21 (76) |
| Symptomatic cryoglobulinemia, n (%) | 5 (100) | 11/20 (55) |
| MoC, n (%) | 5 (100) | 14/20 (70) |
| HCV genotype, n (%)^#^  1  2  3  4 | 3 (60)  2 (40)  -  - | 12 (55)  6 (27)  3 (14)  1 (4) |
| HCV RNA quantitative (copies/ml), median  (range) | 832000 (171700-7817000) | 1232378 (319-13911261) ^§^ |
| Antiviral therapy, n (%) | 5 (100) | 21 (95) |
| Type of antiviral therapy, n (%)  IFN-based DAA-based | 0  5 (100) | 4 (19)  17 (81) |
| Sustained virological response, n (%) | 5 (100) | 17/19 (89) |
| Hematological response to antiviral therapy, n (%)^  CR PR SD  PD | 4 (75)  -  -  1 (25)° | 5 (29)  2 (12)  7 (41)  3 (18) |
| Subsequent hematological treatment, n (%) | - | 8/11 (73) |
| Type of hematological treatment  Rituximab Immunochemotherapy  Chemotherapy | -  -  - | 4 (50)  3 (38)  1 (12) |
| Response to hematological treatment, n(%)  CR PR | -  - | 5 (63)  3 (37) |

**Supplemental Table 3: IGHV and IGLV rearrangements in 27 patients with HCV-associated lymphoproliferative disorders**

| **ID** | **DIAGNOSIS** | **SOURCE** | **IGHV** | **IGHD** | **IGHJ** | **% IDENTITY** | **HCDR3** | **STEREOTYPED SUBSET HCDR3** | **HCDR3 LENGHT** | **E2 HOM** | **RF HOM** | **IGLV** | **IGLJ** | **% IDENTITY** | **LCDR3** | **LCDR3 LENGHT** | **STEREOTYPED SUBSET LCDR3** | **E2 HOM** | **RF HOM** | **TYPE OF AT** | **HEMATOLOGICAL RESPONSE TO AT** |
| --- | --- | --- | --- | --- | --- | --- | --- | --- | --- | --- | --- | --- | --- | --- | --- | --- | --- | --- | --- | --- | --- |
| AG20 | Type II MC | PBMNC | 1-69 | 2-15 | 4 | 95,77 | CAREGRSGYVNPFDYW |  | 14 | NO | YES | Vk3D-20 | 1 | 98.01 | QQYGSSPGT | 9 | 9 AA stereotyped motif | YES | YES | DAA | CR |
| BT01 | SMZL | PBMNC | 1-2 | 7-27 | 4 | 98.94 | CARSGRDQLGIADYW | 13 AA sterotyped subset | 13 | NO | NO | Vk3-15 | 1 | 98 | QHYNNWPPWT | 10 | 10 AA stereotyped motif | NO | YES | DAA | PR |
| CL02 | Type II MC | PBMNC | 1-2 | 7-27 | 4 | 98.94 | CARSGRDQLGIADYW | 13 AA sterotyped subset | 13 | NO | NO | Vk3D-20 | 2 | 98 | QQYGNSPRT | 9 | 9 AA stereotyped motif | YES | YES | DAA | CR |
| EF09 | MZL | PBMNC | 3-30 | 1-26 | 2 | 95.24 | CAKGGPGGIVGLVW |  | 12 | NO | NO | Vk1-8 | 2 | 98.04 | QHYYSYPYT | 9 |  | YES | YES | DAA | SD |
| GB07 | CLL/SLL | PBMNC | 3-7 | 3-22 | 3 | 94.71 | CARGDYYDSSGYFHDAFDVW | 18 AA stereotyped subset | 18 | NO | YES | Vk3-15 | 1 | 98 | QQYNNWPPWT | 10 | 10 AA stereotyped motif | NO | YES | DAA | SD |
| GD17 | Type II MC | PBMNC | 1-69 | 4-17 | 4 | 99.47 | CAREGRGTVTTNPFDYW |  | 15 | NO | YES | Vk3D-20 | 1 | 99.33 | QQYGSSPQT | 9 | 9 AA stereotyped motif | YES | YES | DAA | PD |
| GG14 | Subcutaneous MZL | FFPE | 4-34 | 6-19 | 4 | 87,63 | CARGVCYCSGCFDYW |  | 13 | NO | YES | NA* | NA | NA | NA | NA | NA | NA | NA | DAA | CR |
| GL11 | CLL/SLL | PBMNC | 3-48 | 1-26 | 4 | 100 | CARDGVGGPYW |  | 9 | NO | NO | VL3-21 | 3 | 99.12 | QVWDSSSDHPWV | 12 |  | NO | NO | DAA | PD |
| GM05 | B-NHL NOS | PBMNC | 2-70D | NA | 6 | 99.46 | CARSITLFGVVTNW |  | 12 | NO | NO | Vk3-15 | 1 | 99.12 | QHYNNWPPWT | 10 | 10 AA stereotyped motif | NO | YES | DAA | SD |
| IR08 | FL | PBMNC | 3-7 | NA | NA | NA | NA | NA | NA | NA | NA | Vk3-15 | 1 | NA | QHYNNWPPWT | 10 | 10 AA stereotyped motif | NO | YES | DAA | SD |
| LP19 | Type II MC | PBMNC | 1-69 | 5-24 | 6 | 97.35 | CARKGEMATDPHYYYGMDVW |  | 18 | NO | NO | Vk3D-20 | 2 | 99.33 | QQYGNSPYT | 9 | 9 AA stereotyped motif | YES | YES | DAA | CR |
| MD12 | CLL/SLL | PBMNC | 4-61 | NA | 6 | 100 | CARDPPYCSGGSCYW |  | 13 | YES | YES | Vk1-33 | 5 | 98.04 | QQYDNLPLT | 9 |  | NO | YES | DAA | PD |
| PV-003 | B-NHL NOS | BMMNC | 3-33 | 5-24 | 4 | 94,1 | CARAEGYDDYDYW |  | 11 | NO | NO | NA |  |  | NA | NA | NA | NA | NA | IFN + Ribavirin | NA |
| PV-010 | B-NHL NOS | BMMNC | 3-21 | 3-22 | 4 | 97,57 | CARDRSFDYYDSSGALDYW |  | 17 | NO | NO | NA |  |  | NA | NA | NA | NA | NA | IFN | NA |
| PV-013 | B-NHL NOS | BMMNC | 3-30-3 | 3-10 | 4 | 100 | CARGGRDLLKFDYW |  | 12 | NO | NO | NA |  |  | NA | NA | NA | NA | NA | DAA | PD |
| PV-043 | B-NHL NOS | BMMNC | 4-59 | 3-3 | 6 | 99,3 | CARHQYDFWSGYQASGYGMDVW |  | 20 | NO | NO | NA |  |  | NA | NA | NA | NA | NA | IFN | NA |
| PV-050 | B-NHL NOS | BMMNC | 1-69 | 6-13 | 4 | 90,71 | CAREGFSEKQLTNPIDLW |  | 16 | NO | NO | NA |  |  | NA | NA | NA | NA | NA | IFN + Ribavirin | CR |
| PV-071 | B-NHL NOS | PBMNC | 3-74 | 3-16 | 5 | 90,28 | CVKGGPYGNNWFDSW |  | 13 | NO | NO | NA |  |  | NA | NA | NA | NA | NA | DAA | CR |
| RL18 | Type II MC | PBMNC | 1-69 | 3-22 | 4 | 97.35 | CARGFSPLGDSSGYYYAYW |  | 17 | NO | YES | Vk3D-20 | 2 | 97.37 | QQYGSSPYT | 9 | 9 AA stereotyped motif | YES | YES | DAA | CR |
| RV04 | MZL | PBMNC | 2-5 | 6-13 | 2 | 95.16 | CAHRPPFSSSWYPQWYFDFW |  | 18 | NO | NO | Vk1-5 | 1 | 97.03 | QQYNTYST | 8 |  | NO | NO | DAA | SD |
| SC06 | B-NHL NOS | PBMNC | 3-7 | 3-22 | 3 | 95.24 | CVRGDYDDSSGSYSDAFDIW | 18 AA stereotyped subset | 18 | NO | YES | NA |  |  | NA | NA | NA | NA | NA | DAA | PR |
| ST13 | B-NHL NOS | PBMNC | 4-61 | 3-16 | 4 | 95.16 | CARAERGLGRFDHW |  | 12 | NO | NO | Vk4-1 | 1 | 96.67 | QQYYNTPWT | 9 |  | NO | NO | DAA | SD |
| TM10 | CLL/SLL | PBMNC | 3-33 | 3-10 | 4 | 86.77 | CAKDSRYDGSGSLTNW |  | 14 | NO | NO | NA |  |  | NA | NA | NA | NA | NA | NA | NA |
| VS03 | SMZL | PBMNC | 1-69 | 4-23 | 4 | 93.12 | CAREGKGNDNGGNPFDYW |  | 16 | NO | NO | Vk3D-20 | 4 | 88.35 | QQYGTSPLT | 9 | 9 AA stereotyped motif | YES | YES | DAA | CR |

**Supplemental Table 4: Full annotation and variant allele frequencies (VAFs) for each mutation mutation in 27 patients with hepatitis C virus-associated lymphoproliferative disorders**

| **ID** | **DIAGNOSIS** | **SOURCE*** | **FUNCTION** | **CHR** | **START** | **END** | **REF** | **ALT** | **VAF** | **% CLONAL CELL** | **VAF ADJUSTED*** | **DEPTH** | **GENE** | **TRANSCRIPT** | **EXON** | **C.** | **P.** | **PATHWAY°** |
| --- | --- | --- | --- | --- | --- | --- | --- | --- | --- | --- | --- | --- | --- | --- | --- | --- | --- | --- |
| AG20 | Type II MC | PBMNC | nonsynonymous SNV | 22 | 41566411 | 41566411 | T | C | 0,4406 | 5 | 0,02203 | 985 | EP300 | NM_001429 | 27 | c.T4288C | p.Y1430H | Chromatin organization |
| AG20 | Type II MC | PBMNC | nonsynonymous SNV | 4 | 187540958 | 187540958 | G | A | 0,4976 | 5 | 0,02488 | 2663 | FAT1 | NM_005245 | 10 | c.C6782T | p.T2261M | Cell communication |
| AG20 | Type II MC | PBMNC | nonsynonymous SNV | 19 | 16436805 | 16436805 | A | C | 0,0267 | 5 | 0,001335 | 1085 | KLF2 | NM_016270 | 2 | c.A854C | p.K285T | NF-kappa B signalling |
| BT01 | SMZL | PBMNC | nonsynonymous SNV | 7 | 2984153 | 2984153 | C | T | 0,0292 | 64 | 0,018688 | 1269 | CARD11 | NM_032415 | 5 | c.G377A | p.G126D | NF-kappa B signalling |
| BT01 | SMZL | PBMNC | nonsynonymous SNV | 4 | 187540974 | 187540974 | C | T | 0,4921 | 64 | 0,314944 | 1835 | FAT1 | NM_005245 | 10 | c.G6766A | p.A2256T | Cell communication |
| BT01 | SMZL | PBMNC | stopgain SNV | 4 | 126239775 | 126239775 | C | T | 0,04 | 64 | 0,0256 | 1902 | FAT4 | NM_001291303 | 1 | c.C2209T | p.Q737X | Cell communication |
| BT01 | SMZL | PBMNC | nonsynonymous SNV | 4 | 153249384 | 153249384 | C | A | 0,0268 | 64 | 0,017152 | 1155 | FBXW7 | NM_033632 | 9 | c.G1394T | p.R465L | Notch signalling |
| BT01 | SMZL | PBMNC | nonsynonymous SNV | 6 | 138198231 | 138198231 | T | G | 0,0599 | 64 | 0,038336 | 852 | TNFAIP3 | NM_001270508 | 6 | c.T824G | p.L275R | NF-kappa B signalling |
| BT01 | SMZL | PBMNC | frameshift_variant | 6 | 138196950 | 138196950 | A | - | 0,06683 | 64 | 0,0427712 | 1295 | TNFAIP3 | NM_001270508 | 4 | c.613delA | p.R205fs | NF-kappa B signalling |
| CL02 | Type II MC | PBMNC | frameshift_variant | 6 | 26184043 | 26184043 | C | - | 0,4388 | 12 | 0,052656 | 2322 | HIST1H2BE | NM_003523 | 1 | c.20delC | p.S7fs | Chromatin organization |
| CL02 | Type II MC | PBMNC | nonsynonymous SNV | 3 | 183209851 | 183209851 | A | G | 0,0349 | 12 | 0,004188 | 2065 | KLHL6 | NM_130446 | 7 | c.T1730C | p.L577P | B cell receptor signalling |
| CL02 | Type II MC | PBMNC | nonsynonymous SNV | 9 | 8319899 | 8319899 | C | T | 0,4976 | 12 | 0,059712 | 2062 | PTPRD | NM_002839 | 45 | c.G5602A | p.G1868R | Cell communication |
| EF09 | MZL | PBMNC | nonsynonymous SNV | 7 | 2979513 | 2979513 | A | G | 0,0604 | 6 | 0,003624 | 1638 | CARD11 | NM_032415 | 6 | c.T734C | p.L245P | NF-kappa B signalling |
| EF09 | MZL | PBMNC | nonsynonymous SNV | 22 | 23230372 | 23230373 | AG | CC | 0,042 | 6 | 0,00252 | 857 | IGLL5 | NM_001178126 | 1 | c.139_140delAGinsCC | p.S47P | B cell receptor signalling |
| EF09 | MZL | PBMNC | nonsynonymous SNV | 19 | 16436813 | 16436813 | C | G | 0,0895 | 6 | 0,00537 | 1447 | KLF2 | NM_016270 | 2 | c.C862G | p.H288D | NF-kappa B signalling |
| FC15 | MZL | FFPE | frameshift_variant | 15 | 93545434 | 93545434 | A | - | 0,0792 | NA |  | 1490 | CHD2 | NM_001271 | 33 | c.4173delA | p.K1391fs | Chromatin organization |
| FC15 | MZL | FFPE | nonsynonymous SNV | 16 | 85942755 | 85942755 | A | C | 0,29288 | NA |  | 1391 | IRF8 | NM_002163 | 3 | c.A334C | p.I112L | Interferon Signalling |
| FC15 | MZL | FFPE | stopgain SNV | 12 | 49436379 | 49436379 | G | C | 0,27275 | NA |  | 1362 | KMT2D | NM_003482 | 27 | c.C5832G | p.Y1944X | Chromatin organization |
| FC15 | MZL | FFPE | frameshift_variant | 1 | 120458454 | 120458455 | - | G | 0,2646 | NA |  | 1325 | NOTCH2 | NM_024408 | 34 | c.6890dupC | p.R2298fs | Notch signalling |
| FC15 | MZL | FFPE | nonsynonymous SNV | 19 | 42599749 | 42599749 | C | T | 0,05673 | NA |  | 1389 | POU2F2 | NM_001207025 | 10 | c.G854A | p.R285K | Transcriptional regulation |
| FC15 | MZL | FFPE | frameshift_variant | 3 | 176743309 | 176743309 | A | - | 0,2761 | NA |  | 1340 | TBL1XR1 | NM_024665 | 16 | c.1522delT | p.C508fs | Chromatin organization |
| FC15 | MZL | FFPE | frameshift_variant | 6 | 138200148 | 138200161 | AGCCTGCCTCCAGG | - | 0,24357 | NA |  | 1408 | TNFAIP3 | NM_001270508 | 7 | c.1567_1580delGCCTGCCTCCAGGA | p.A523fs | NF-kappa B signalling |
| GB07 | CLL/SLL | PBMNC | stopgain SNV | 15 | 93545542 | 93545542 | G | T | 0,0847 | 46 | 0,038962 | 1535 | CHD2 | NM_001271 | 33 | c.G4273T | p.E1425X | Chromatin organization |
| GB07 | CLL/SLL | PBMNC | nonsynonymous SNV | 22 | 41574829 | 41574829 | A | G | 0,4925 | 46 | 0,22655 | 1864 | EP300 | NM_001429 | 31 | c.A7114G | p.M2372V | Chromatin organization |
| GB07 | CLL/SLL | PBMNC | nonsynonymous SNV | 5 | 89990446 | 89990446 | C | T | 0,4775 | 46 | 0,21965 | 2333 | GPR98 | NM_032119 | 33 | c.C7873T | p.R2625C | G protein Signalling |
| GB07 | CLL/SLL | PBMNC | nonsynonymous SNV | 3 | 183273281 | 183273281 | A | C | 0,0545 | 46 | 0,02507 | 2369 | KLHL6 | NM_130446 | 1 | c.T161G | p.L54R | B cell receptor signalling |
| GG14 | Subcutaneous MZL | FFPE | nonsynonymous SNV | 12 | 92539204 | 92539204 | C | A | 0,393 | NA |  | 1464 | BTG1 | NM_001731 | 1 | c.G108T | p.Q36H | Cell cycle |
| GG14 | Subcutaneous MZL | FFPE | nonsynonymous SNV | 7 | 2977603 | 2977603 | A | C | 0,3436 | NA |  | 1259 | CARD11 | NM_032415 | 8 | c.T1081G | p.Y361D | NF-kappa B signalling |
| GG14 | Subcutaneous MZL | FFPE | stopgain SNV | 6 | 29910399 | 29910399 | G | A | 0,28 | NA |  | 1307 | HLA-A | NM_002116 | 1 | c.G69A | p.W23X | Antigen processing and presentation |
| GG14 | Subcutaneous MZL | FFPE | nonsynonymous SNV | 7 | 151860463 | 151860463 | C | T | 0,026 | NA |  | 2220 | KMT2C | NM_170606 | 43 | c.G10199A | p.R3400H | Chromatin organization |
| GG14 | Subcutaneous MZL | FFPE | stopgain SNV | 12 | 49445440 | 49445440 | C | A | 0,1441 | NA |  | 1347 | KMT2D | NM_003482 | 10 | c.G2026T | p.E676X | Chromatin organization |
| GG14 | Subcutaneous MZL | FFPE | nonsynonymous SNV | 2 | 61145601 | 61145601 | G | A | 0,036 | NA |  | 1590 | REL | NM_002908 | 7 | c.G713A | p.R238H | Ras signalling |
| GG14 | Subcutaneous MZL | FFPE | nonsynonymous SNV | 3 | 176756171 | 176756171 | C | T | 0,52103 | NA |  | 1250 | TBL1XR1 | NM_024665 | 11 | c.G977A | p.S326N | Chromatin organization |
| GG14 | Subcutaneous MZL | FFPE | splice_donor_variant | 6 | 138197303 | 138197303 | G | - | 0,44887 | NA |  | 1580 | TNFAIP3 | NM_001270508 | 5 | c.805+1delG |  | NF-kappa B signalling |
| GL11 | CLL/SLL | PBMNC | nonsynonymous SNV | 4 | 187534286 | 187534286 | A | C | 0,4265 | 79 | 0,336935 | 952 | FAT1 | NM_005245 | 13 | c.T9440G | p.V3147G | Cell communication |
| GL11 | CLL/SLL | PBMNC | nonsynonymous SNV | 2 | 198267483 | 198267483 | C | A | 0,4111 | 79 | 0,324769 | 3600 | SF3B1 | NM_012433 | 14 | c.G1874T | p.R625L | Spliceosome |
| GM05 | B-NHL NOS | PBMNC | nonsynonymous SNV | 3 | 176750830 | 176750830 | C | G | 0,064 | 19 | 0,01216 | 1548 | TBL1XR1 | NM_024665 | 14 | c.G1345C | p.A449P | Chromatin organization |
| IR08 | FL | PBMNC | nonsynonymous SNV | 16 | 30905199 | 30905199 | C | T | 0,3623 | 7 | 0,025361 | 1380 | BCL7C | NM_001286526 | 1 | c.G67A | p.A23T | Chromatin organization |
| IR08 | FL | PBMNC | nonsynonymous SNV | 7 | 151962282 | 151962282 | G | A | 0,0253 | 7 | 0,001771 | 1503 | KMT2C | NM_170606 | 8 | c.C1025T | p.A342V | Chromatin organization |
| IR08 | FL | PBMNC | nonsynonymous SNV | 9 | 8521327 | 8521327 | G | C | 0,4982 | 7 | 0,034874 | 1367 | PTPRD | NM_002839 | 20 | c.C911G | p.A304G | Cell communication |
| IR08 | FL | PBMNC | nonsynonymous SNV | 10 | 43615083 | 43615083 | C | T | 0,546 | 7 | 0,03822 | 978 | RET | NM_020975 | 14 | c.C2497T | p.R833C | RET signalling |
| LP19 | Type II MC | PBMNC | frameshift_variant | 9 | 139390649 | 139390650 | AG | - | 0,05305 | 19 | 0,0100795 | 1591 | NOTCH1 | NM_017617 | 34 | c.7541_7542delCT | p.P2514fs | Notch signalling |
| LP19 | Type II MC | PBMNC | stopgain SNV | 6 | 138196014 | 138196014 | C | T | 0,0528 | 19 | 0,010032 | 1667 | TNFAIP3 | NM_001270508 | 3 | c.C328T | p.Q110X | NF-kappa B signalling |
| MD12 | CLL/SLL | PBMNC | nonsynonymous SNV | 15 | 66727443 | 66727443 | T | G | 0,135 | 81 | 0,10935 | 2549 | MAP2K1 | NM_002755 | 2 | c.T159G | p.F53L | MAPK signalling |
| MD12 | CLL/SLL | PBMNC | nonsynonymous SNV | 2 | 198266611 | 198266611 | C | T | 0,3337 | 81 | 0,270297 | 1747 | SF3B1 | NM_012433 | 16 | c.G2225A | p.G742D | Spliceosome |
| MD12 | CLL/SLL | PBMNC | nonsynonymous SNV | 1 | 216462695 | 216462695 | G | A | 0,4867 | 81 | 0,394227 | 2176 | USH2A | NM_007123 | 11 | c.C1898T | p.S633L | Protein binding |
| MDA16 | MZL | PBMNC | nonsynonymous SNV | 4 | 126241248 | 126241248 | C | G | 0,4959 | 5 | 0,024795 | 2083 | FAT4 | NM_001291303 | 1 | c.C3682G | p.Q1228E | Cell communication |
| MDA16 | MZL | PBMNC | nonsynonymous SNV | 7 | 151970855 | 151970856 | GT | AA | 0,06318 | 5 | 0,003159 | 5079 | KMT2C | NM_170606 | 7 | c.946_947delACinsTT | p.T316F | Chromatin organization |
| PV-003 | B-NHL NOS | BMMNC | frameshift_variant | 9 | 139390649 | 139390650 | AG | - | 0,262 | 34 | 0,08908 | 1497 | NOTCH1 | NM_017617 | 34 | c.7541_7542delCT | p.P2514fs | Notch signalling |
| PV-003 | B-NHL NOS | BMMNC | frameshift_variant | 6 | 138192649 | 138192658 | GAAAACGAAC | - | 0,02269 | 34 | 0,0077146 | 1597 | TNFAIP3 | NM_001270508 | 2 | c.286_295delAAAACGAACG | p.K96fs | NF-kappa B signalling |
| PV-010 | B-NHL NOS | BMMNC | nonsynonymous SNV | 11 | 102201850 | 102201850 | G | A | 0,4039 | 15 | 0,060585 | 1478 | BIRC3 | NM_001165 | 6 | c.G1202A | p.R401K | NF-kappa B signalling |
| PV-010 | B-NHL NOS | BMMNC | nonsynonymous SNV | 12 | 113496173 | 113496173 | C | A | 0,061 | 15 | 0,00915 | 2673 | DTX1 | NM_004416 | 1 | c.C176A | p.S59Y | Notch signalling |
| PV-010 | B-NHL NOS | BMMNC | stopgain SNV | 4 | 126239880 | 126239880 | C | T | 0,0497 | 15 | 0,007455 | 2754 | FAT4 | NM_001291303 | 1 | c.C2314T | p.Q772X | Cell communication |
| PV-010 | B-NHL NOS | BMMNC | nonsynonymous SNV | 22 | 23237737 | 23237737 | A | C | 0,5106 | 15 | 0,07659 | 2446 | IGLL5 | NM_001178126 | 3 | c.A508C | p.S170R | B cell receptor signalling |
| PV-010 | B-NHL NOS | BMMNC | frameshift_variant | 1 | 120458434 | 120458435 | - | T | 0,0586 | 15 | 0,00879 | 2609 | NOTCH2 | NM_024408 | 34 | c.6910dupA | p.I2304fs | Notch signalling |
| PV-010 | B-NHL NOS | BMMNC | nonsynonymous SNV | 1 | 144931218 | 144931218 | G | C | 0,2208 | 15 | 0,03312 | 3932 | PDE4DIP | NM_001002811 | 1 | c.C491G | p.P164R | Cell communication |
| PV-013 | B-NHL NOS | BMMNC | stopgain SNV | 6 | 41903731 | 41903731 | G | A | 0,4 | 51 | 0,204 | 2000 | CCND3 | NM_001136017 | 5 | c.C583T | p.Q195X | Cell cycle |
| PV-013 | B-NHL NOS | BMMNC | nonsynonymous SNV | 7 | 151945228 | 151945228 | G | A | 0,0438 | 51 | 0,022338 | 1690 | KMT2C | NM_170606 | 14 | c.C2291T | p.S764F | Chromatin organization |
| PV-013 | B-NHL NOS | BMMNC | nonsynonymous SNV | 10 | 43613908 | 43613908 | A | T | 0,4254 | 51 | 0,216954 | 1456 | RET | NM_020975 | 13 | c.A2372T | p.Y791F | RET signalling |
| PV-013 | B-NHL NOS | BMMNC | nonsynonymous SNV | 1 | 16262741 | 16262741 | C | T | 0,5356 | 51 | 0,273156 | 1450 | SPEN | NM_015001 | 11 | c.C10006T | p.R3336W | Notch signalling |
| PV-013 | B-NHL NOS | BMMNC | frameshift_variant | 17 | 7579372 | 7579373 | GC | - | 0,6952 | 51 | 0,354552 | 1479 | TP53 | NM_000546 | 4 | c.314_315del | p.105_105del | p53 signalling |
| PV-043 | B-NHL NOS | BMMNC | nonsynonymous SNV | 3 | 187442794 | 187442794 | G | A | 0,4883 | 25 | 0,122075 | 1710 | BCL6 | NM_001706 | 9 | c.C1912T | p.R638C | Transcriptional regulation |
| PV-043 | B-NHL NOS | BMMNC | nonsynonymous SNV | 19 | 16436813 | 16436813 | C | T | 0,0953 | 25 | 0,023825 | 724 | KLF2 | NM_016270 | 2 | c.C862T | p.H288Y | NF-kappa B signalling |
| PV-050 | B-NHL NOS | BMMNC | nonsynonymous SNV | 5 | 89948255 | 89948255 | A | G | 0,2 | 15 | 0,03 | 1500 | GPR98 | NM_032119 | 19 | c.A3509G | p.Y1170C | G protein Signalling |

| PV-050 | B-NHL NOS | BMMNC | stopgain SNV | 1 | 2488165 | 2488165 | T | A | 0,1677 | 15 | 0,025155 | 1610 | TNFRSF14 | NM_003820 | 1 | c.T62A | p.L21X | NF-kappa B signalling |
| --- | --- | --- | --- | --- | --- | --- | --- | --- | --- | --- | --- | --- | --- | --- | --- | --- | --- | --- |
| PV-071 | B-NHL NOS | PBMNC | nonsynonymous SNV | 7 | 151932949 | 151932949 | C | A | 0,0517 | 50 | 0,02585 | 1722 | KMT2C | NM_170606 | 16 | c.G2722T | p.G908C | Chromatin organization |
| PV-071 | B-NHL NOS | PBMNC | nonsynonymous SNV | 3 | 38182025 | 38182025 | G | T | 0,4395 | 50 | 0,21975 | 3238 | MYD88 | NM_001172567 | 3 | c.G649T | p.V217F | Toll-like receptor signalling |
| PV-071 | B-NHL NOS | PBMNC | nonsynonymous SNV | 6 | 37139203 | 37139203 | G | C | 0,3295 | 50 | 0,16475 | 1217 | PIM1 | NM_002648 | 4 | c.G543C | p.E181D | Jak-STAT Signalling |
| PV-071 | B-NHL NOS | PBMNC | nonsynonymous SNV | 17 | 7577133 | 7577133 | T | C | 0,1165 | 50 | 0,05825 | 1614 | TP53 | NM_000546 | 8 | c.A805G | p.S269G | p53 signalling |
| PV-072 | B-NHL NOS | BMMNC | nonsynonymous SNV | 3 | 38181988 | 38181988 | G | T | 0,5353 | 92 | 0,492476 | 1457 | MYD88 | NM_001172567 | 3 | c.G612T | p.L204F | Toll-like receptor signalling |
| PV-072 | B-NHL NOS | BMMNC | frameshift_variant | 6 | 138199573 | 138199574 | - | A | 0,03254 | 92 | 0,0299368 | 864 | TNFAIP3 | NM_001270508 | 7 | c.992dupA | p.D331fs | NF-kappa B signalling |
| RV04 | MZL | PBMNC | nonsynonymous SNV | 6 | 29911302 | 29911302 | G | A | 0,3305 | 19 | 0,062795 | 593 | HLA-A | NM_002116 | 3 | c.G601A | p.E201K | Antigen processing and presentation |
| SC06 | B-NHL NOS | PBMNC | nonsynonymous SNV | 4 | 126239839 | 126239839 | C | T | 0,467 | 21 | 0,09807 | 2210 | FAT4 | NM_001291303 | 1 | c.C2273T | p.A758V | Cell communication |
| SC06 | B-NHL NOS | PBMNC | nonsynonymous SNV | 6 | 27860548 | 27860548 | G | C | 0,071 | 21 | 0,01491 | 1818 | HIST1H2AM | NM_003514 | 1 | c.C380G | p.A127G | Chromatin organization |
| SC06 | B-NHL NOS | PBMNC | nonsynonymous SNV | 3 | 183273174 | 183273174 | G | A | 0,1027 | 21 | 0,021567 | 2055 | KLHL6 | NM_130446 | 1 | c.C268T | p.L90F | B cell receptor signalling |
| SC06 | B-NHL NOS | PBMNC | frameshift_variant | 6 | 138200037 | 138200037 | C | - | 0,03954 | 21 | 0,0083034 | 1786 | TNFAIP3 | NM_001270508 | 7 | c.1455delC | p.F485fs | NF-kappa B signalling |
| ST13 | B-NHL NOS | PBMNC | nonsynonymous SNV | 4 | 187549808 | 187549808 | A | G | 0,4674 | 10 | 0,04674 | 2176 | FAT1 | NM_005245 | 8 | c.T4433C | p.I1478T | Cell communication |
| ST13 | B-NHL NOS | PBMNC | nonsynonymous SNV | 13 | 41240285 | 41240285 | G | C | 0,1905 | 10 | 0,01905 | 2100 | FOXO1 | NM_002015 | 1 | c.C65G | p.S22W | Transcriptional regulation |
| ST13 | B-NHL NOS | PBMNC | nonsynonymous SNV | 1 | 216465694 | 216465694 | G | C | 0,4158 | 10 | 0,04158 | 1763 | USH2A | NM_007123 | 10 | c.C1663G | p.L555V | Protein binding |
| TM10 | CLL/SLL | PBMNC | nonsynonymous SNV | 9 | 8518271 | 8518271 | C | T | 0,4802 | 79 | 0,379358 | 1316 | PTPRD | NM_002839 | 21 | c.G1120A | p.A374T | Cell communication |
| TM10 | CLL/SLL | PBMNC | stopgain SNV | 3 | 176771619 | 176771619 | A | T | 0,3813 | 79 | 0,301227 | 695 | TBL1XR1 | NM_024665 | 4 | c.T146A | p.L49X | Chromatin organization |
| TM10 | CLL/SLL | PBMNC | nonsynonymous SNV | 7 | 98606099 | 98606099 | A | C | 0,1314 | 79 | 0,103806 | 1279 | TRRAP | NM_001244580 | 69 | c.A10811C | p.K3604T | Transcriptional regulation |
| VS03 | SMZL | PBMNC | nonsynonymous SNV | 12 | 4385250 | 4385250 | C | G | 0,4562 | 17 | 0,077554 | 594 | CCND2 | NM_001759 | 2 | c.C275G | p.P92R | Cell cycle |
| VS03 | SMZL | PBMNC | nonsynonymous SNV | 4 | 126371133 | 126371133 | T | C | 0,1381 | 17 | 0,023477 | 637 | FAT4 | NM_001291303 | 9 | c.T8968C | p.Y2990H | Cell communication |
| VS03 | SMZL | PBMNC | nonsynonymous SNV | 3 | 176744204 | 176744204 | T | A | 0,0894 | 17 | 0,015198 | 1492 | TBL1XR1 | NM_024665 | 15 | c.A1475T | p.N492I | Chromatin organization |

# Supplemental table 5: Correlation of LCDR3 stereotypes with mutational status.

**Genes mutated in KCDR3 stereotyped subsets**

**Genes unmutated in KCDR3 stereotyped**

**subsets**

| **Pt.** | **Diagnosis** | **IGHV** | **LCDR3** | **RF**  **hom** | **E2**  **hom** | **Chromatin organization** | | | | | | **NFkB** | | | **Cell communication** | | | **BCR** | | **Notch** | | **RET** | **G**  **prot** | **Cell cycl** | **Tran scr.** | | **MAP K** | **Splice** | **Prot bind** | **HLA** |
| --- | --- | --- | --- | --- | --- | --- | --- | --- | --- | --- | --- | --- | --- | --- | --- | --- | --- | --- | --- | --- | --- | --- | --- | --- | --- | --- | --- | --- | --- | --- |
| **BCL7** | | | | | | | **CHD** | **EP** | **HIST1** | **TBL1** | **KMT CARD KLF2 TNFA** | | | | **FAT** | **FAT4** | **PTP** | **IGL** | **KLH** | **Notch** | **FBX** | **RET** | **GPR98** | **CCND2** | **FOXO1** | | **MAP2** | **SF3B1** | **USH2** | **HLAA** |
|  |  |  |  |  |  | **C** | **2** | **300** | **H2BE** | **XR1** | **2C** | **11** | | **IP3** | **1** |  | **RD** | **L5** | **L6** | **1** | **W7** |  |  |  |  |  | **K1** |  | **A** |  |
| AG20 | **Type II MC** | **1-69** | **QQYGSSPGT** | **Y** | **Y** | 0 | 0 | 1 | 0 | 0 | 0 | 0 | 1 | 0 | 1 | 0 | 0 | 0 | 0 | 0 | 0 | 0 | 0 | 0 |  | 0 | 0 | 0 | 0 | 0 |
| CL02 | **Type II MC** | **1-2** | **QQYGNSPRT** | **Y** | **Y** | 0 | 0 | 0 | 1 | 0 | 0 | 0 | 0 | 0 | 0 | 0 | 1 | 0 | 1 | 0 | 0 | 0 | 0 | 0 |  | 0 | 0 | 0 | 0 | 0 |
| LP19 | **Type II MC** | **1-69** | **QQYGNSPYT** | **Y** | **Y** | 0 | 0 | 0 | 0 | 0 | 0 | 0 | 0 | 1 | 0 | 0 | 0 | 0 | 0 | 1 | 0 | 0 | 0 | 0 |  | 0 | 0 | 0 | 0 | 0 |
| VS03 | **SMZL** | **1-69** | **QQYGTSPLT** | **Y** | **Y** | 0 | 0 | 0 | 0 | 1 | 0 | 0 | 0 | 0 | 0 | 1 | 0 | 0 | 0 | 0 | 0 | 0 | 0 | 1 |  | 0 | 0 | 0 | 0 | 0 |
| EF09 | **MZL** | **3-30** | **QHYYSYPYT** | **Y** | **Y** | 0 | 0 | 0 | 0 | 0 | 0 | 1 1 | | 0 | 0 | 0 | 0 | 1 | 0 | 0 | 0 | 0 | 0 | 0 |  | 0 | 0 | 0 | 0 | 0 |
| BT01 | **SMZL** | **1-2** | **QHYNNWPPWT** | **Y** | **N** | 0 | 0 | 0 | 0 | 0 | 0 | 1 | 0 | 1 | 1 | 1 | 0 | 0 | 0 | 0 | 1 | 0 | 0 | 0 |  | 0 | 0 | 0 | 0 | 0 |
| GM05 | **NHL NOS** | **2-70D** | **QHYNNWPPWT** | **Y** | **N** | 0 | 0 | 0 | 0 | 1 | 0 | 0 | 0 | 0 | 0 | 0 | 0 | 0 | 0 | 0 | 0 | 0 | 0 | 0 |  | 0 | 0 | 0 | 0 | 0 |
| IR08 | **FL** | **3-7** | **QHYNNWPPWT** | **Y** | **N** | 1 | 0 | 0 | 0 | 0 | 1 | 0 | 0 | 1 | 0 | 0 | 1 | 0 | 0 | 0 | 0 | 1 | 0 | 0 |  | 0 | 0 | 0 | 0 | 0 |
| GB07 | **CLL/SLL** | **3-7** | **QQYNNWPPWT** | **Y** | **N** | 0 | 1 | 1 | 0 | 0 | 0 | 0 | 0 | 0 | 0 | 0 | 0 | 0 | 1 | 0 | 0 |  | 1 | 0 |  | 0 | 0 | 0 | 0 | 0 |
|  |  |  |  |  |  |  |  |  |  |  |  |  | |  |  |  |  |  |  |  |  |  |  |  |  |  |  |  |  |  |
| GL11 | **CLL/SLL** | **3-48** | **QVWDSSSDHPWV** | **N** | **N** | 0 | 0 | 0 | 0 | 0 | 0 | 0 0 | | 0 | 1 | 0 | 0 | 0 | 0 | 0 | 0 | 0 | 0 | 0 |  | 0 | 0 | 1 | 0 | 0 |
| MD12 | **CLL/SLL** | **4-61** | **QQYDNLPLT** | **Y** | **N** | 0 | 0 | 0 | 0 | 0 | 0 | 0 0 | | 0 | 0 | 0 | 0 | 0 | 0 | 0 | 0 | 0 | 0 | 0 |  | 0 | 1 | 1 | 1 | 0 |
| ST13 | **NHL NOS** | **4-61** | **QQYYNTPWT** | **N** | **N** | 0 | 0 | 0 | 0 | 0 | 0 | 0 0 | | 0 | 1 | 0 | 0 | 0 | 0 | 0 | 0 | 0 | 0 | 0 |  | 1 | 0 | 0 | 1 | 0 |
| RV04 | **MZL** | **2-5** | **QQYNTYST** | **N** | **N** | 0 | 0 | 0 | 0 | 0 | 0 | 0 0 | | **0** | 0 | 0 | 0 | 0 | 0 | 0 | 0 | 0 | 0 | 0 |  | 0 | 0 | 0 | 0 | 1 |

**A *vs* B, p= 0.6623 A *vs* C, p=0.0172 B *vs* C, p=0.0026**

**A+B *vs* C, p= 0.0018**

**A *vs* B, p=1.00**

**A *vs* C, p=0.0083 B *vs* C, p=0.0083**

**A+B *vs* C, p=0.0002**
